# Radiomics: Current Applications and Future Directions

**DOI:** 10.1002/mco2.70773

**Published:** 2026-05-23

**Authors:** Jiangbo Shao, Meng Wei, Ke Li, Guangchao Lv, Kai Liu, Ye Guo

**Affiliations:** ^1^ Ultrasound Diagnostic Center The First Hospital of Jilin University Jilin China; ^2^ Department of General Gynecology II, Obstetrics and Gynecology Center The First Hospital of Jilin University Jilin China; ^3^ Department of Emergency Medicine The First Hospital of Jilin University Jilin China; ^4^ Department of Thoracic Surgery II, Department of Lung Transplantation, Organ Transplantation Center The First Hospital of Jilin University Jilin China; ^5^ Department of Hand and Foot Surgery Orthopedics Center The First Hospital of Jilin University Jilin China; ^6^ Cancer Center The First Hospital of Jilin University Jilin China

**Keywords:** artificial intelligence, clinical translation, multi‐omics fusion, pan‐cancer, radiomics

## Abstract

Radiomics enables high‐throughput extraction of quantitative imaging features to decode tumor phenotypes and biological behaviors, representing a transformative noninvasive tool for precision oncology. In recent years, radiomics has rapidly evolved from static feature analysis to dynamic multi‐dimensional assessment, and it has been widely explored in various solid tumors, yet its pan‐cancer generalization, biological interpretability, and clinical translation still face prominent bottlenecks. Cancer remains the leading cause of global mortality, and solid tumors account for more than 90% of adult malignant cases, while conventional medical imaging and invasive biopsies have inherent limitations in reflecting tumor heterogeneity and dynamic evolution. This review outlines the unified technical pipeline of radiomics across solid tumors, highlights cancer‐specific imaging considerations, and summarizes standardization strategies for multi‐center, multi‐scanner, and multi‐cancer heterogeneity. We systematically review pan‐cancer clinical applications covering early detection, molecular characterization, treatment response prediction, and prognostic stratification, with lung cancer as a paradigmatic example while integrating evidence from breast, colorectal, liver, glioma, and prostate cancers. We also discuss multi‐omics integration, biological interpretability, and translational bottlenecks including domain shift and reproducibility crisis. Finally, we prospect cutting‐edge directions including foundation models, causal inference, and federated learning to advance generalizable and clinically actionable radiomics toward routine clinical practice.

## Graphical abstract

Radiomics enables high‐throughput extraction of quantitative imaging features to decode tumor phenotypes across solid tumors. This panoramic review summarizes its technical pipeline, standardization strategies, pan‐cancer clinical applications, and translational bottlenecks. It further highlights future directions including foundation models, causal inference, and federated learning toward clinically actionable precision oncology.

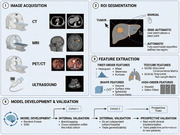

## 1 Introduction

Cancer remains the leading cause of global mortality, with solid tumors accounting for more than 90% of adult malignant cases [1, 2]. Despite remarkable advances in surgery, chemotherapy, targeted therapy, and immune checkpoint blockade, clinical oncology still faces persistent and unresolved challenges [3, 4]. These include low rates of early detection leading to most patients being diagnosed at advanced stages, profound inter‐ and intra‐tumoral heterogeneity that drives highly variable treatment responses and prognosis, the inherent limitations of invasive tissue biopsies in capturing spatial and temporal molecular evolution, and the substantial lag of traditional size‐based efficacy evaluation that relies on response evaluation criteria in solid tumors (RECIST) criteria [5, 6]. Conventional medical imaging provides essential anatomical and morphological information but is largely limited to qualitative visual interpretation, failing to quantify the subtle microscale biological characteristics that determine tumor behavior and treatment sensitivity [7, 8].

Radiomics is defined as the systematic extraction and high‐throughput mining of massive quantitative features from medical images to generate interpretable and noninvasive biomarkers, bridging macroscopic imaging phenotypes and underlying molecular and cellular mechanisms [9, 10, 11]. Since its conceptualization, radiomics has undergone rapid evolution from simple static whole‐tumor feature analysis to dynamic longitudinal monitoring and refined subregional heterogeneity characterization [12, 13, 14]. In recent years, it has further integrated with artificial intelligence, multi‐omics, and liquid biopsy to form a comprehensive multi‐dimensional clinical decision‐making system [15, 16]. Compared with invasive detection approaches, radiomics offers unique advantages including noninvasiveness, repeatable whole‐tumor sampling, and cost‐effectiveness, demonstrating universal and transformative value in precision oncology across nearly all solid tumor types.

The developmental trajectory of radiomics can be divided into three key phases [17, 18]. The first phase (2012–2018) was dominated by traditional static feature analysis, focusing on first‐order statistics, texture, and shape features derived mainly from CT images. The second phase (2019–2022) witnessed the rise of dynamic and subregional approaches, represented by delta radiomics for longitudinal change quantification and habitat radiomics for intratumoral subregion decomposition. The third phase (2023–present) marks the era of multi‐technology integration, driven by deep learning, multi‐omics fusion, large‐scale data collaboration, and regulatory translation. Several high‐quality recent reviews have standardized the methodological framework and clarified translational pathways, laying a critical foundation for the development of pan‐cancer radiomics [19].

This review adopts a broad pan‐cancer perspective covering the most prevalent and clinically significant solid tumors, including lung, breast, colorectal, hepatocellular carcinoma, glioma, and prostate cancer [20, 21]. We applied consistent and rigorous literature selection criteria: priority was given to multi‐center prospective studies, high‐quality systematic reviews and meta‐analyses, and clinical practice guidelines; single‐center small‐sample exploratory studies without external validation, non‐oncology studies, and purely technical reports with limited clinical relevance were excluded [22, 23]. The review is structured around a coherent technology–application–translation framework, aiming to address five core scientific questions [24, 25]: (1) the general technical pipeline and cancer‐specific imaging adaptations of pan‐cancer radiomics; (2) the shared principles and heterogeneous characteristics of clinical applications across different tumor types; (3) the mechanisms and value of multi‐omics integration and biological interpretability; (4) the universal and tumor‐specific bottlenecks restricting clinical translation; and (5) the future directions toward generalizable, causal, and clinically usable radiomics.

This panoramic review provides a comprehensive and up‐to‐date overview of radiomics from technical fundamentals to pan‐cancer applications and translational prospects. By using lung cancer as the most extensively studied and mature paradigm while systematically integrating high‐level evidence from multiple other solid tumors, we clearly distinguish between generalizable radiomic principles and cancer‐specific findings [26, 27, 28]. We critically highlight current limitations, evidence gaps, and methodological flaws, and propose a rational and actionable roadmap for accelerating the credible clinical implementation of radiomics in daily oncology practice [29, 30].

## 2 Core Technical Pipeline and Standardization Across Cancers

Radiomics follows a broadly unified technical workflow across all solid tumor types, yet the unique anatomical locations, tissue compositions, biological behaviors, and routine clinical imaging protocols of different cancers demand tailored methodological optimization [31, 32, 33]. This section systematically introduces the general radiomics pipeline, cancer‐specific imaging considerations, key standardization strategies for multi‐source heterogeneity, emerging advanced technical branches, and the comparative roles of deep learning and handcrafted features in pan‐cancer scenarios.

### 2.1 General Workflow: Image Acquisition, Segmentation, and Feature Extraction

The core radiomics pipeline consists of four sequential and interdependent steps: standardized imaging data acquisition, accurate and reproducible region of interest (ROI) segmentation, high‐dimensional feature extraction and dimensionality reduction, and rigorous model construction and validation [34, 35]. Each step directly determines the reliability, reproducibility, and clinical applicability of the final radiomic model.

Imaging acquisition constitutes the foundational step for robust radiomic analysis [36, 37, 38]. Across most solid tumors, computed tomography (CT) remains the most widely applied modality owing to its high spatial resolution, widespread clinical availability, and cost efficiency. Positron emission tomography (PET/CT) adds crucial metabolic and functional information that complements anatomical details. Magnetic resonance imaging (MRI) provides superior soft‐tissue contrast and rich multi‐parameter functional sequences including diffusion, perfusion, and hepatobiliary phase imaging. Ultrasound is commonly used for superficial organs, interventional guidance, and serial follow‐up. To ensure reproducible feature calculation, scanning parameters such as slice thickness, reconstruction kernel, tube voltage, and window settings must be standardized and documented in detail [39, 40, 41].

ROI segmentation is a critical determinant of feature stability and can be categorized into three types with distinct performance profiles [42, 43, 44]. Manual segmentation, performed by experienced radiologists, offers high accuracy for complex lesions but suffers from low efficiency and significant inter‐observer variability. Semi‐automatic segmentation, assisted by thresholding or region‐growing algorithms, improves efficiency while maintaining reasonable consistency. Fully automatic segmentation based on deep learning models achieves the highest efficiency and reproducibility, making it suitable for large‐scale multi‐center studies [45]. The choice of segmentation strategy should be determined by tumor type, lesion size, boundary clarity, sample size, and study purpose [46, 47].

**FIGURE 1:**
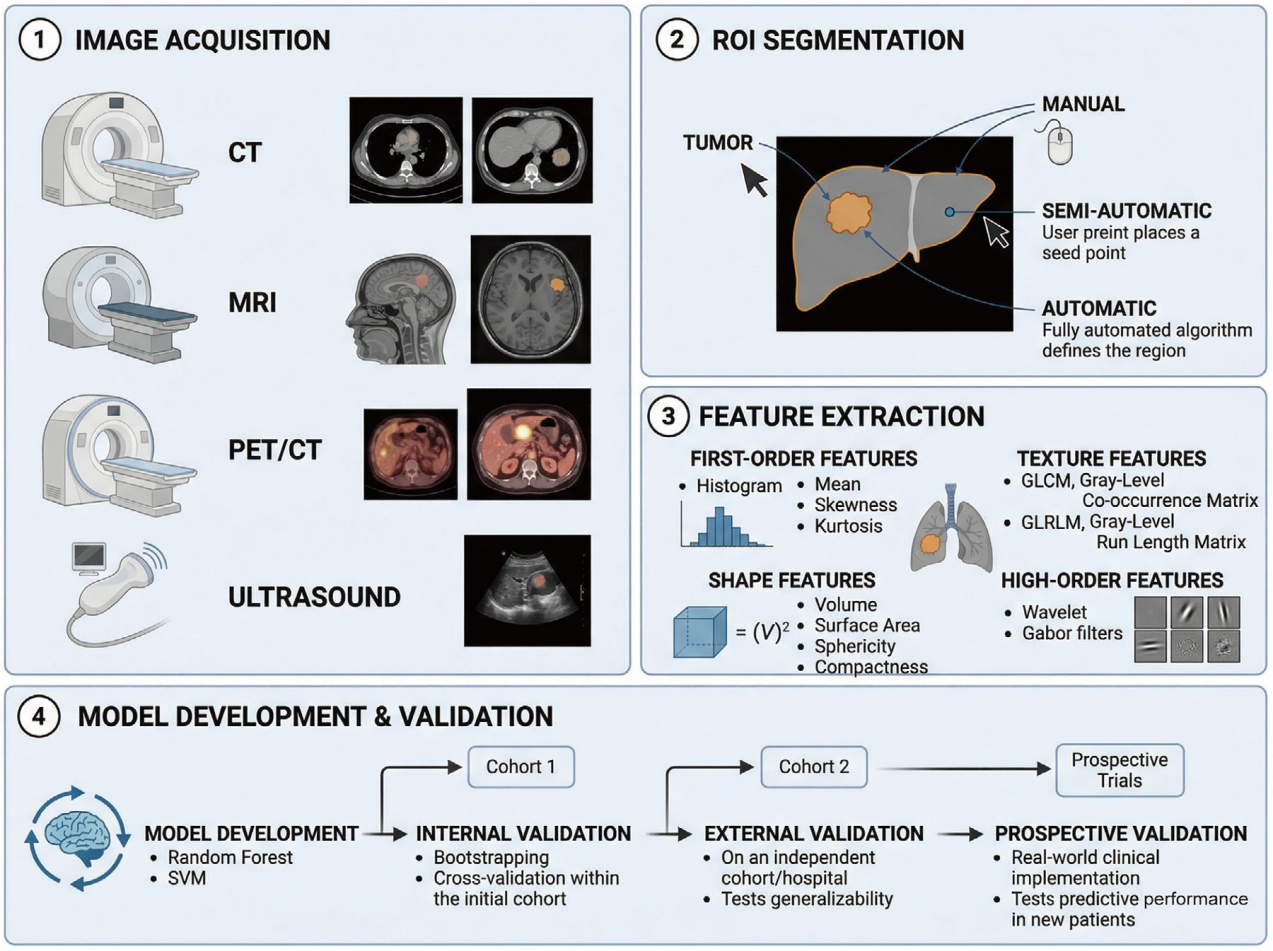
Schematic overview of the unified pan‐cancer radiomics technical pipeline. The workflow comprises four core steps: (1) standardized image acquisition using CT, MRI, PET/CT, or ultrasound; (2) ROI segmentation including manual, semi‐automatic, and fully automatic approaches; (3) high‐throughput feature extraction covering first‐order statistics, texture, shape, and high‐order filtered features; and (4) dimensionality reduction, model construction, and rigorous validation including internal, external, and prospective validation.

Feature extraction generates a large set of quantitative descriptors that capture invisible tumor characteristics [48]. These include first‐order features reflecting pixel intensity distribution, texture features depicting spatial pixel correlations, shape features describing geometric morphology, and higher order features enhanced by wavelet or Laplacian of Gaussian filtering. Given the high dimensionality and redundancy of extracted features, feature selection is essential to eliminate noise and multicollinearity. Common methods include correlation analysis, LASSO regression, minimum redundancy maximum relevance (mRMR), and recursive feature elimination, all of which improve model generalization, interpretability, and clinical practicality (Figure 1).

### 2.2 Cancer‐Specific Imaging Considerations

Different solid tumors exhibit distinct anatomical sites, tissue density, vascularity, border characteristics, and typical growth patterns, which directly determine the optimal imaging modalities, segmentation strategies, and meaningful feature sets in radiomics [49]. These cancer‐specific properties must be carefully considered to ensure model performance and clinical relevance.

For lung cancer, CT represents the undisputed dominant imaging modality in radiomic analysis [50]. To retain the subtle structural characteristics of ground‐glass nodules and small peripheral lesions that are critical for early detection and risk stratification, thin‐slice reconstruction (≤1.25 mm) and standardized lung window settings are essential prerequisites for reliable radiomic feature extraction. Segmentation consistency is especially critical in this malignancy, given the high prevalence of small, ill‐defined, and peripherally located lesions; even minor variations in ROI delineation can introduce substantial instability and reduce the reproducibility and predictive performance of radiomic models.

For breast cancer, mammography, ultrasound, and dynamic contrast‐enhanced MRI are employed in a complementary manner to capture distinct aspects of tumor pathophysiology [51]. Mammography excels in the detection of microcalcifications associated with early malignant transformation [52]. Ultrasound provides high sensitivity for identifying small lesions in radiographically dense breast parenchyma. Meanwhile, texture features and kinetic enhancement curves derived from dynamic contrast‐enhanced MRI reflect underlying angiogenesis, cellularity, and stromal fibrosis, all of which correlate closely with tumor invasiveness, molecular subtype, and treatment response.

For colorectal cancer, pelvic MRI and abdominal CT constitute the standard imaging framework for radiomic assessment [53]. Abdominal CT enables evaluation of distant metastatic spread and colonic wall thickening, whereas pelvic MRI is indispensable for characterizing local tumor invasion, mesorectal fascia involvement, and regional lymph node status. These anatomical and radiomic features directly inform surgical planning, neoadjuvant treatment strategies, and prognostic stratification, making standardized imaging acquisition critical for clinical translation.

For hepatocellular carcinoma, contrast‐enhanced CT and MRI represent the first‐line imaging modalities for radiomic analysis [54]. Functional and perfusion‐related radiomic features derived from these modalities reflect characteristic hemodynamic changes in hypervascular tumors, as well as background liver function reserve and parenchymal disease status. Together, these imaging features enable accurate lesion characterization, risk stratification, and assessment of treatment feasibility, supporting personalized clinical decision‐making.

For glioma, brain MRI serves as the gold standard imaging modality for radiomic profiling [55]. Multi‐parametric MRI sequences, including T1‐weighted, T2‐weighted, FLAIR, and diffusion‐weighted imaging, capture key pathophysiological features including tumor infiltration, peritumoral edema, blood–brain barrier disruption, and cellularity. Radiomic features derived from these sequences enable noninvasive assessment of tumor grade, molecular subtype, and prognosis, with substantial implications for surgical planning and adjuvant therapy.

For prostate cancer, multi‐parametric MRI stands as the cornerstone of noninvasive radiomic diagnosis [56]. Combined T2‐weighted, diffusion‐weighted, and dynamic contrast‐enhanced sequences enable reliable detection of clinically significant lesions, particularly in the transition zone. Radiomic models built upon these data improve risk stratification, reduce unnecessary prostate biopsies, and guide targeted sampling and treatment selection, thereby refining overall patient management.

These cancer‐specific imaging characteristics determine the modality selection, appropriate segmentation algorithms, feature prioritization, and validation strategies, representing key factors that distinguish successful, clinically relevant radiomic models from technically sound but non‐translatable research.

### 2.3 Standardization Challenges: Multi‐Center, Multi‐Scanner and Multi‐Cancer

Heterogeneity represents the most critical bottleneck restricting the clinical translation of pan‐cancer radiomics [57]. This heterogeneity arises from three major interconnected sources and severely impairs model reproducibility and generalization.

First, device and parameter heterogeneity stems from differences in scanner manufacturers, tube voltage, tube current, slice thickness, reconstruction algorithms, and post‐processing settings [58]. These differences can cause 15%–20% variation in feature values for the same lesion, directly distorting model performance. Second, segmentation heterogeneity arises from inter‐observer differences, semi‐automatic versus automatic methods, and ROI boundary definitions, with shape and texture features being particularly sensitive [59]. Third, feature definition heterogeneity results from divergent software implementations, leading to non‐comparable feature values that prevent cross‐study validation and meta‐analysis [60].

**FIGURE 2:**
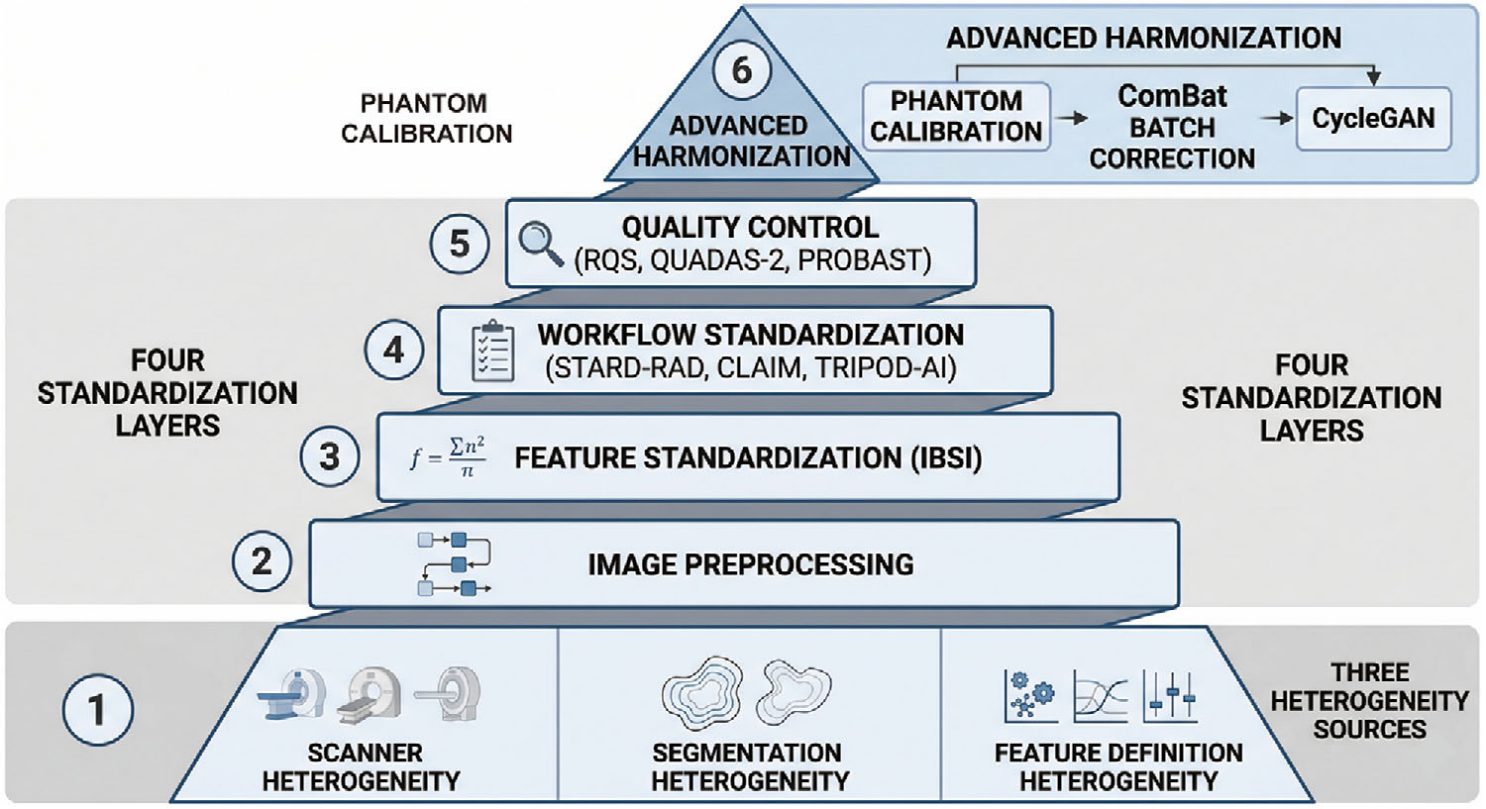
Hierarchical standardization and harmonization strategy for multi‐center, multi‐scanner pan‐cancer radiomics. Three major sources of heterogeneity are addressed: scanner/parameter variability, segmentation inconsistency, and feature definition differences. A multi‐layered solution includes image preprocessing, IBSI‐aligned feature standardization, workflow standardization following STARD‐RAD, CLAIM, and TRIPOD‐AI guidelines, and strict quality control using RQS, QUADAS‐2, and PROBAST. Advanced harmonization methods include phantom calibration, ComBat batch correction, and CycleGAN domain adaptation.

To address these challenges, a multi‐layered standardization strategy is required. Image preprocessing includes voxel spacing normalization, intensity clipping, artifact reduction, and noise suppression [61]. Feature standardization follows unified definitions provided by the Image Biomarker Standardization Initiative (IBSI), combined with Z‐score or Min‐Max normalization. Workflow standardization adheres to reporting guidelines including Standards for Reporting of Diagnostic Accuracy for Radiomics (STARD‐RAD), Checklist for Artificial Intelligence in Medical Imaging (CLAIM), and Transparent Reporting of a Multivariable Prediction Model for Individual Prognosis or Diagnosis for Artificial Intelligence (TRIPOD‐AI). Quality control is enforced using tools such as the Radiomics Quality Score (RQS), Quality Assessment of Diagnostic Accuracy Studies 2 (QUADAS‐2), and Prediction Model Risk of Bias Assessment Tool (PROBAST). Advanced harmonization methods including phantom‐based calibration, ComBat batch correction, and Cycle‐Consistent Generative Adversarial Networks (CycleGAN) domain adaptation have further emerged as effective approaches to reduce multi‐center and multi‐scanner bias (Figure 2).

### 2.4 Emerging Branches: Dynamic, Delta, and Subregional Radiomics

Traditional static radiomics captures only baseline tumor phenotypes, which is insufficient to reflect the dynamic biological changes induced by treatment or disease progression [62]. Several emerging branches have significantly expanded the capability and clinical value of radiomics by enabling dynamic and spatially refined analysis.

Delta radiomics quantifies the changes in radiomic features between two or more time points, such as pre‐treatment, mid‐treatment, and post‐treatment, which are critical for evaluating treatment efficacy [63]. This branch focuses on dynamic tumor feature variations over time rather than just static baseline characteristics. By capturing treatment‐induced tumor evolution—including changes in size, density, texture, and vascularity—delta radiomics enables early prediction of treatment response, dynamic patient risk stratification, and early warning of adverse events, often weeks ahead of conventional imaging‐detected anatomical changes. This early capability helps clinicians adjust regimens timely for non‐responders, improving outcomes and reducing treatment‐related toxicity [64].

Habitat radiomics uses clustering algorithms to divide tumors into biologically meaningful subregions (proliferative, necrotic, hypoxic, and invasive zones), each linked to distinct molecular profiles and treatment sensitivities [65]. Unlike traditional radiomics that treats tumors as homogeneous entities, it decodes intratumoral heterogeneity—a major driver of treatment resistance and recurrence. Different subregions respond differently to therapies, so independent subregion analysis provides precise tumor biology insights, aiding personalized treatment design.

Dynamic radiomics integrates longitudinal multi‐time‐point imaging data (collected during treatment and follow‐up) to construct real‐time adaptive risk models that evolve with tumor behavior. It goes beyond delta radiomics by incorporating continuous rather than discrete temporal changes, enabling dynamic monitoring of progression and response [66]. Adaptive models update with new imaging data, offering real‐time risk assessments to guide timely treatment adjustments, enhancing prognosis accuracy and personalized management.

To address heterogeneity limiting radiomics clinical translation, a multi‐layered standardization strategy is needed [67]. Image preprocessing includes voxel spacing normalization (consistent spatial resolution), intensity clipping (eliminating artifact‐induced extreme values), artifact reduction, and noise suppression—laying the foundation for reliable feature extraction.

Feature standardization follows the Image Biomarker Standardization Initiative (IBSI) unified definitions, combined with Z‐score or Min‐Max normalization to eliminate imaging parameter impacts [68]. Workflow standardization adheres to STARD‐RAD, CLAIM, and TRIPOD‐AI guidelines, ensuring research transparency and reproducibility.

Quality control uses tools like RQS (methodological quality), QUADAS‐2 (diagnostic bias), and PROBAST (prediction model bias) [69]. Advanced harmonization methods (phantom‐based calibration, ComBat batch correction, and CycleGAN domain adaptation) effectively reduce multi‐center/multi‐scanner bias, improving model reproducibility and generalization.

Together, these advanced branches enhance the biological relevance, predictive accuracy, and clinical utility of radiomics, especially in the context of treatment response monitoring, personalized prognosis, and adaptive therapy design [70].

### 2.5 Deep Learning‐Based Radiomics Versus Handcrafted Features

Handcrafted radiomics and deep learning‐based radiomics represent two complementary yet distinct analytical paradigms that are widely applied in pan‐cancer research [71]. Handcrafted features are predefined mathematical descriptors including intensity statistics, texture indices, shape parameters, and filter‐derived higher order values, which are constructed based on prior knowledge of image characteristics. These features offer high transparency, good interpretability, and reliable performance in small‐sample or single‐center studies, making them particularly suitable for biological annotation and mechanistic validation with histological or molecular markers. However, handcrafted features have limited capacity to capture high‐dimensional, implicit, and cross‐modal patterns, which may restrict performance in complex multi‐scenario or large‐scale datasets [72].

In contrast, deep learning‐based radiomics relies on convolutional neural networks (CNNs) or other deep architectures to automatically learn hierarchical feature representations directly from raw images, without manual feature engineering [73]. This data‐driven paradigm excels in fusing multi‐modal information, capturing abstract imaging patterns, and delivering superior performance in large‐sample, multi‐center, and cross‐cancer tasks. Nevertheless, deep learning models are often regarded as “black boxes” with limited interpretability, requiring large annotated datasets and high computational costs, which hinder their biological validation and clinical transparency [74].

In current pan‐cancer radiomics practice, a hybrid strategy that integrates handcrafted features and deep learning representations has become the preferred consensus. This combined approach leverages the interpretability and biological grounding of handcrafted features while utilizing the powerful feature learning capability of deep learning models [75, 76]. With the assistance of explainable artificial intelligence (XAI) techniques, the hybrid framework enhances the interpretability of deep learning outputs and strengthens the predictive power of handcrafted features, achieving an optimal balance between model accuracy, robustness, biological interpretability, and clinical translatability across diverse cancer types.

## 3 Multi‐Omics Integration: A Pan‐Cancer Perspective

Radiomics alone captures macroscopic phenotypic information of tumors, including morphological characteristics such as size, shape, and texture, as well as functional features reflected in medical images [77]. However, this single‐dimensional analysis can only observe the surface manifestations of tumors and cannot penetrate into the intrinsic biological mechanisms behind imaging phenotypes. In contrast, multi‐omics integration—combining radiomics with genomics, transcriptomics, proteomics, and metabolomics—enables deep decoding of the underlying molecular pathways, gene expression programs, and cellular processes that drive imaging phenotypes. These molecular‐level insights help explain why different tumors exhibit distinct imaging features, bridging the gap between macroscopic imaging manifestations and microscopic biological mechanisms.

This multi‐omics integration further forms a comprehensive “imaging–molecule–clinical” pan‐cancer decision support system [78]. By fusing imaging data, molecular data, and clinical information, this system not only significantly enhances the accuracy of disease diagnosis, treatment response prediction, and prognosis evaluation but also strengthens the biological interpretability of radiomic features. It allows clinicians to gain a more comprehensive understanding of tumor biology, thereby providing more scientific and targeted guidance for clinical decision‐making, personalized treatment, and follow‐up management [79].

### 3.1 Radiogenomics: Linking Imaging Phenotypes to Molecular Pathways Across Cancers

Radiogenomics is an interdisciplinary field that aims to establish quantitative relationships between radiomic features and genomic, transcriptomic, or epigenetic profiles [80]. By exploring the inherent biological basis of imaging phenotypes, it transforms radiomic features from purely statistical correlates into biologically interpretable biomarkers, thereby strengthening the translational value of radiomics in clinical oncology.

Across multiple types of solid tumors, consistent radiogenomic associations have been recognized [81]. Proliferation‐related pathways, including E2F targets and G2M checkpoint regulators, show strong correlations with textural heterogeneity and entropy, reflecting the degree of intratumoral structural disorder. Immune‐related pathways, such as IFN‐γ signaling and T‐cell activation, are closely associated with PET metabolic parameters and intratumoral metabolic heterogeneity, linking imaging features to the tumor immune microenvironment. Angiogenesis pathways, represented by VEGF signaling, are correspondingly manifested in contrast enhancement patterns and perfusion‐related features, providing noninvasive indicators of tumor vascularity [82].

Cancer‐specific radiogenomic signatures have also been well documented [83]. EGFR‐mutant lung cancers typically present with a high proportion of ground‐glass opacity and low entropy. HER2‐positive breast cancers exhibit characteristic textural heterogeneity. IDH‐mutant gliomas display distinct MRI signal patterns. These conserved cross‐cancer and tumor‐specific associations validate the biological foundations of radiomics and support its clinical application in noninvasive molecular characterization of malignant tumors.

### 3.2 Radiomics and Liquid Biopsy (ctDNA, Exosomes)

Liquid biopsy, encompassing circulating tumor DNA (ctDNA), circulating tumor cells (CTCs), and exosomes, permits real‐time and noninvasive surveillance of dynamic molecular alterations during tumor evolution and therapeutic intervention [84, 85]. As a minimally invasive approach, it captures longitudinal changes in tumor genetics and burden that may not be fully reflected by static imaging alone. The synergistic integration of radiomics and liquid biopsy therefore establishes a robust multi‐modal monitoring framework, whose performance surpasses that of either modality used in isolation.

Combining mid‐treatment ctDNA kinetics with delta radiomic features enables continuous, dynamic risk stratification and early prediction of disease progression, often preceding detectable anatomical changes [86]. Joint analysis of radiomic parameters and ctDNA characteristics also markedly enhances the sensitivity of minimal residual disease (MRD) detection, allowing reliable prediction of tumor recurrence months in advance of conventional imaging modalities. Furthermore, the integration of CTC counts with radiomic markers of intratumoral heterogeneity improves the precision of metastatic risk evaluation, offering more robust stratification for high‐risk patient populations [87, 88].

This integrated synergistic strategy has demonstrated broad applicability across multiple common malignancies, including lung, colorectal, breast, and liver cancers [89, 90]. By compensating for the inherent limitations of single‐modal detection, this combined approach provides a more comprehensive and accurate depiction of tumor burden, biological activity, and spatiotemporal heterogeneity, thereby supporting more refined clinical decision‐making in precision oncology.

### 3.3 Cross‐Modal Image Fusion (PET/CT, PET/MRI, etc.)

Different imaging modalities provide distinct and complementary information regarding tumor anatomy, function, metabolism, and cellularity [91]. Each modality captures unique aspects of tumor pathophysiology, and no single imaging technique can fully represent the complex biological characteristics of malignant lesions. Cross‐modal fusion effectively integrates such multi‐dimensional information, thereby significantly enhancing the stability, predictive power, and clinical applicability of radiomic models [92].

Fusion of CT and PET/CT combines high‐resolution anatomical details with quantitative metabolic activity derived from PET imaging [93]. This integrated strategy improves the accuracy of pulmonary nodule characterization, tumor staging, and treatment response evaluation by jointly evaluating structural changes and metabolic alterations. Similarly, fusion of MRI and PET/CT leverages the excellent soft‐tissue resolution of MRI and the metabolic sensitivity of PET, offering substantial advantages in the evaluation of brain, liver, pelvic, and retroperitoneal tumors. In addition, multi‐parametric MRI fusion, which integrates multiple MRI sequences, enables more refined risk stratification in prostate cancer and glioma by capturing diverse tissue properties including cellularity, perfusion, and microstructure.

Cross‐modal integration can be implemented at multiple levels [94]. Feature‐level fusion concatenates quantitative radiomic features from different modalities to construct a comprehensive feature space [95]. Decision‐level fusion combines predictions from individual models through weighted integration to improve overall performance. Deep learning‐based cross‐modal attention mechanisms automatically learn intrinsic correlations between different modalities. Each of these fusion strategies enhances model robustness, diagnostic accuracy, and generalization ability across a wide spectrum of cancer types [96].

### 3.4 Biological Interpretability: Toward a Pan‐Cancer Imaging Omics Atlas

Poor biological interpretability continues to represent a major obstacle to the widespread clinical adoption and regulatory approval of radiomic models. Despite their promising predictive performance in retrospective studies, many radiomic signatures lack clear mechanistic links to underlying tumor biology, limiting their translational potential. To address this critical limitation, multiple complementary strategies are currently being developed to transform radiomics from a purely correlational analytical tool into a mechanistically interpretable technology with solid biological grounding [97, 97].

Advanced XAI techniques, such as Grad‐CAM and LIME, enable the visualization of critical image regions and quantitative features that predominantly drive model predictions, thereby improving transparency and clinical intuitiveness [98]. Pathology–imaging spatial registration and alignment further validate radiomic features by correlating them with well‐established histological markers, including Ki‐67, CD8, and PD‐L1, bridging macroscopic imaging traits with microscopic tissue characteristics. Causal inference methodologies are also employed to distinguish genuine causal relationships from spurious statistical correlations between radiomic features and clinical outcomes.

**FIGURE 3:**
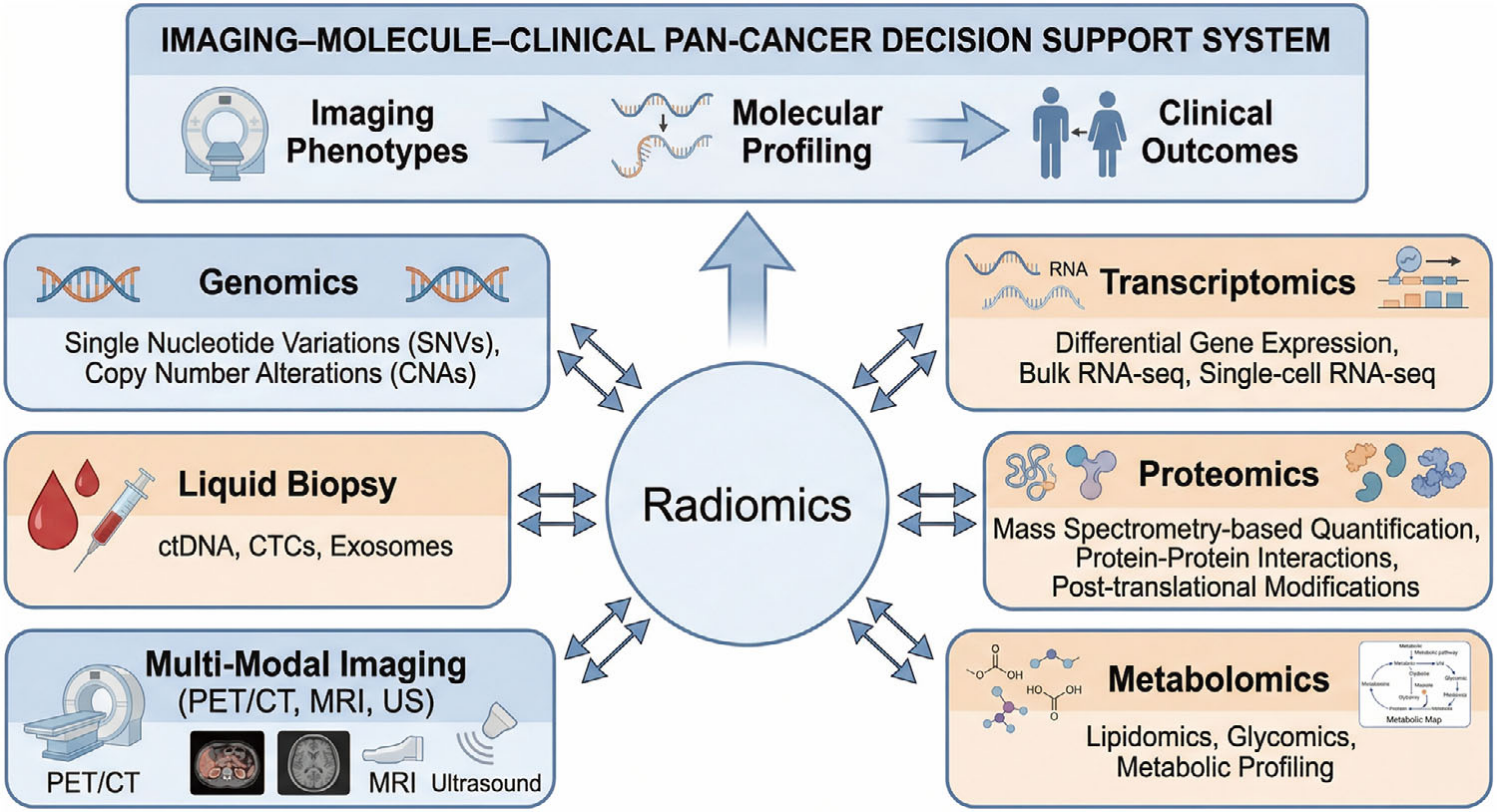
Multi‐omics integration framework for pan‐cancer radiomics. Radiomics serves as a central hub connecting genomics, transcriptomics, proteomics, metabolomics, liquid biopsy (ctDNA, CTCs, exosomes), and multi‐modal imaging. This integrated strategy builds a comprehensive imaging–molecule–clinical decision support system to improve biological interpretability and clinical performance across solid tumors.

In parallel, the ongoing development of a pan‐cancer imaging omics atlas seeks to establish unified, standardized mappings connecting radiomic features, molecular pathways, and histological phenotypes across multiple cancer types [99]. This atlas will serve as a shared reference resource that systematically annotates imaging features with corresponding biological and clinical annotations, enabling consistent interpretation, cross‐cancer generalization, and reliable validation of radiomic models. The successful construction and implementation of such a comprehensive atlas will greatly facilitate the robust and credible clinical translation of radiomic models into routine oncology practice (Figure 3).

## 4 Clinical Applications: Problem‐Oriented Across Major Cancer Types

**FIGURE 4:**
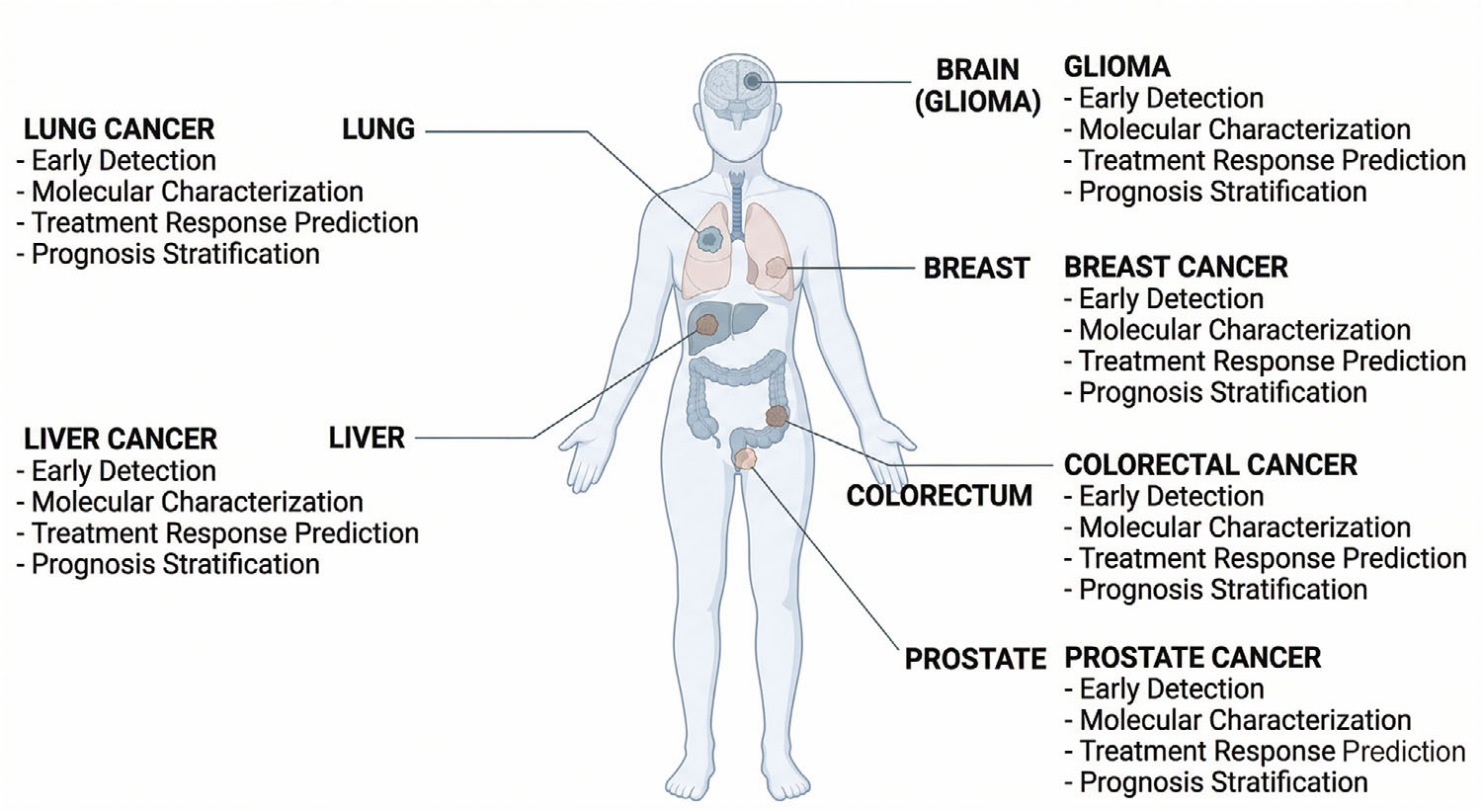
Pan‐cancer clinical applications of radiomics across major solid tumors. Radiomics has been widely applied in lung, breast, colorectal, hepatocellular carcinoma, glioma, and prostate cancer. Core clinical scenarios include early detection and screening, non‐invasive molecular characterization, treatment response prediction, and prognosis and recurrence risk stratification.

Radiomic biomarkers enable consistent and translatable clinical decision support across many solid tumors by addressing unifying clinical challenges in oncology, rather than evaluating applications within single disease entities [100]. Evidence from lung, breast, colorectal, liver, prostate cancers, and gliomas is synthesized to identify shared mechanistic and predictive patterns, while also highlighting distinct cancer‐specific features. This pan‐cancer structure clarifies widely generalizable principles alongside tumor‐unique phenotypic traits, yielding a cohesive evidence base that supports robust, scalable clinical implementation across diverse oncological settings (Figure 4).

### 4.1 Early Detection and Screening

Early detection represents the most impactful strategy to reduce cancer‐related mortality and improve long‐term survival across malignancies, and radiomics offers a noninvasive, quantitative, and highly scalable approach to augment the performance of conventional cancer screening programs [101]. By extracting high‐dimensional quantitative features from clinical imaging, radiomics enables more sensitive and specific risk stratification in populations undergoing routine screening [102].

In lung cancer, CT‐based radiomics enables high‐performance classification of benign versus malignant pulmonary nodules, with particular utility in ground‐glass opacities and small subcentimeter lesions that remain diagnostically challenging for visual radiologic assessment alone [103]. For breast cancer, radiomic analysis of mammographic and ultrasound images reduces false‐positive recall rates and improves the identification of early invasive cancers that may be overlooked on subjective interpretation [104]. In colorectal cancer, radiomic features derived from CT colonography aid in stratifying the malignant potential of colorectal polyps, supporting more targeted intervention [105]. In liver tumors, contrast‐enhanced radiomic profiling improves discrimination between dysplastic nodules and early‐stage hepatocellular carcinoma, facilitating timely intervention [106]. For prostate cancer, multi‐parametric MRI radiomics enhances the detection of clinically significant disease while reducing unnecessary prostate biopsies [107].

A unifying pan‐cancer principle is that radiomics improves early detection by quantifying subtle textural, morphological, and functional imaging features beyond the limits of human visual perception [108]. Cancer‐specific variations predominantly manifest in the selection of imaging modalities, the relative importance of distinct radiomic features, and the integration of radiomic outputs into existing clinical diagnostic pathways.

### 4.2 Noninvasive Molecular Characterization

Radiomics enables noninvasive inference of molecular subtypes, driver mutations, and key therapeutic targets, thereby minimizing the need for serial invasive biopsies and mitigating the inherent limitations of tissue sampling bias [109]. By capturing macroscopic imaging correlates of underlying molecular alterations, radiomic profiling offers a repeatable and whole‐lesion assessment of tumor molecular status that cannot be fully achieved by conventional histologic sampling.

In lung cancer, radiomic models predict EGFR, KRAS, ALK, and ROS1 alterations as well as PD‐L1 expression with AUC values consistently exceeding 0.85, supporting robust noninvasive genotyping. In breast cancer, radiomic features show significant correlations with ER, PR, and HER2 status, as well as intrinsic molecular subtypes. In colorectal cancer, radiomics facilitates the prediction of RAS/RAF mutations and microsatellite instability, both of which direct clinical therapeutic decisions. In glioma, radiomic signatures reliably identify IDH mutation, 1p/19q codeletion, and MGMT methylation status, which define major clinicomolecular subgroups. In prostate cancer, radiomic profiles are strongly associated with the Ki‐67 proliferation index and established molecular risk stratification systems.

A unifying pan‐cancer principle is that imaging phenotypes directly reflect underlying genomic and transcriptomic aberrations [110]. Cancer‐specific divergence emerges primarily from variations in driver gene prevalence, mutation spectrum, and the distinct imaging signatures associated with lineage‐specific molecular programs [111].

### 4.3 Treatment Response Prediction

Radiomics enables early, dynamic, and noninvasive prediction of treatment response in diverse malignancies, often preceding detectable changes in tumor size by weeks—filling a critical unmet need in oncology, where timely assessment of therapeutic efficacy directly guides clinical decision‐making. By capturing subtle, quantitative changes in tumor phenotype over time, radiomic profiling provides an earlier readout of treatment effect than conventional anatomical imaging, enabling prompt adjustment of therapeutic strategies to optimize outcomes.

In chemoradiotherapy, delta radiomics—quantifying longitudinal changes in radiomic features—predicts treatment response 4–6 weeks earlier than the standard RECIST criteria across lung, esophageal, and rectal cancers, facilitating early identification of non‐responders [112]. In immunotherapy, radiomic features effectively distinguish between “hot” (immunogenically active) and “cold” (immunologically quiescent) tumors, predict objective response rates, and forecast the risk of immune‐related adverse events across a spectrum of solid tumors [113]. In targeted therapy, radiomic models reliably predict treatment efficacy and the emergence of early acquired resistance in lung, breast, and colorectal cancers, supporting personalized adjustment of targeted agents [114]. In anti‐angiogenic therapy, radiomic features reflect tumor vascular density, permeability, and heterogeneity, serving as noninvasive surrogates for treatment response in liver and lung cancers [115].

A shared pan‐cancer principle underlying these applications is that quantitative alterations in radiomic features precede macroscopic anatomical tumor shrinkage, offering a sensitive early marker of therapeutic effect [116]. Cancer‐specific differences in this context primarily stem from variations in standard treatment regimens, tumor response kinetics, and the sets of high‐value radiomic features that best capture treatment‐induced phenotypic changes for each malignancy.

### 4.4 Prognosis and Recurrence Risk Stratification

Radiomics enables robust and comprehensive prognostic modeling by synergistically integrating clinical variables, quantitative imaging features, and molecular characteristics, frequently achieving superior stratification performance compared with traditional tumor staging systems [95, 112]. By capturing intratumoral heterogeneity and spatial complexity across the entire lesion, radiomic signatures provide a more holistic assessment of tumor biological behavior and individual patient risk.

In lung cancer, radiomic‐based models reliably predict overall survival, progression‐free survival, and the risk of local or distant recurrence, with C‐statistics consistently exceeding 0.90 [117]. In breast cancer, radiomic profiling facilitates accurate risk stratification for tumor recurrence and aids in the rational selection of adjuvant therapeutic strategies [118]. In colorectal cancer, radiomic signatures predict both metastatic potential and long‐term postoperative survival outcomes [119]. In glioma, radiomic analysis supports prognostic stratification and helps differentiate pseudoprogression from true disease progression, a critical distinction in neuro‐oncological management [120]. In liver cancer, radiomic features provide effective prediction of tumor recurrence following surgical resection or locoregional ablation [121].

A unifying pan‐cancer principle is that radiomic heterogeneity serves as a quantitative surrogate for tumor aggressiveness, invasive potential, and metastatic propensity [122]. Cancer‐specific variations manifest mainly in distinct recurrence patterns, divergent definitions of high‐risk radiomic features, and different clinical intervention thresholds tailored to each tumor type.

### 4.5 Cancer‐Specific Deep Dives (Summary Table)

To facilitate a clear and systematic comparison of evidence level, clinical maturity, and translational status across major cancer types, we summarize the core application scenarios, evidence quality, clinical readiness, and prevailing challenges in a structured summary table (Table 1). This table quantitatively and qualitatively evaluates the translational stage of radiomics in each tumor type, with standardized grading of evidence level and clinical maturity [123].

**TABLE 1:** Pan‐cancer radiomics: Application scenarios, evidence level, clinical maturity, and core challenges.

Cancer type	Core clinical application scenarios	Evidence level	Clinical maturity	Core challenges	References
Lung cancer	Nodule classification; EGFR/KRAS/PD‐L1 prediction; immunotherapy/chemoradiotherapy response; recurrence and prognosis stratification	M+P	CR	CT parameter heterogeneity; ground‐glass nodule segmentation; limited prospective validation	[50, 117, 124]
Breast cancer	Benign/malignant differentiation; molecular subtype prediction; neoadjuvant response; recurrence risk stratification	M+R	CR	Multi‐modal inconsistency; ultrasound feature variability; poor interpretability	[51, 80, 118]
Colorectal cancer	Polyp risk assessment; RAS/MSI prediction; staging; liver metastasis risk; chemoradiotherapy response	M+R	CR	MRI protocol variability; peristalsis artifacts; limited liquid biopsy integration	[53, 105, 119]
Hepatocellular carcinoma	Early nodule diagnosis; grading; TACE/ablation response; recurrence prediction	M+R	CR	Liver parenchyma interference; contrast protocol differences; MVI prediction difficulty	[54, 121]
Glioma	IDH/1p19q/MGMT prediction; grading; pseudoprogression differentiation; prognosis	M+R	RM	Motion artifacts; peritumor segmentation instability; protocol heterogeneity	[55, 120]
Prostate cancer	Clinically significant cancer detection; molecular risk; biopsy guidance; recurrence stratification	M+P	CR	mpMRI standardization; ROI variability; insufficient real‐world data	[56, 107]

*^Note:^* Evidence level: P = prospective; M = multi‐center; R = retrospective; E = exploratory. Clinical maturity: CR = clinically ready; RM = research‐oriented.

Lung cancer remains the most mature field with the highest level of multi‐center prospective evidence and clear clinical readiness, owing to standardized imaging protocols, large patient cohorts, and extensive validation [124]. Breast, colorectal, hepatocellular carcinoma, and prostate cancers exhibit moderate‐to‐high clinical maturity with growing multi‐center evidence, but are limited by multi‐modal inconsistency, protocol variability, or insufficient real‐world data [125]. Glioma shows robust research‐oriented performance but faces greater challenges from anatomical complexity, motion artifacts, and peritumor segmentation variability [126]. Collectively, this table highlights the progressive gradient of clinical translation and identifies common and cancer‐specific barriers that must be addressed to promote widespread clinical adoption.

## 5 Clinical Translation Bottlenecks in the Multi‐Cancer Setting

Despite the substantial advancements achieved in radiomics research across solid tumors in recent years, the clinical translation and real‐world application of pan‐cancer radiomics models remain significantly constrained [127, 128]. A multitude of interconnected barriers—spanning technical, clinical, methodological, and regulatory domains—have hindered the translation of most radiomic signatures into routine clinical practice, limiting their utility in standard oncology care. These bottlenecks are not confined to specific cancer types but are universal across malignancies; however, they are further exacerbated in the pan‐cancer context, where increased biological heterogeneity (across different tumor types) and technical variability (across imaging platforms and protocols) amplify the challenges of standardization and clinical validation.

Technical barriers include inconsistencies in imaging acquisition parameters, variability in feature extraction methodologies, and a lack of standardized preprocessing workflows, all of which compromise the reproducibility and generalizability of radiomic models [129, 130]. Clinically, the integration of radiomic outputs into existing diagnostic and therapeutic workflows remains cumbersome, with limited awareness among clinicians regarding the optimal application of radiomic insights [131]. Methodologically, gaps persist in the standardization of feature definition, quality control, and cross‐study validation, while regulatory hurdles—including the lack of clear guidelines for radiomic assay validation and clinical implementation—further impede translational progress. Collectively, these interconnected obstacles prevent the widespread adoption of pan‐cancer radiomics, despite its proven potential to enhance clinical decision‐making.

### 5.1 Data Heterogeneity and Lack of External Validation

Data heterogeneity and insufficient external validation constitute the most fundamental obstacles to the clinical translation of pan‐cancer radiomics [132, 133]. Currently, more than 70% of published radiomics studies are single‐center, retrospective, and based on small sample sizes (often fewer than 200 patients), with narrow and selective inclusion criteria. Such designs introduce substantial selection bias, model overfitting, and inflated performance estimates that poorly reflect real‐world clinical scenarios.

Worryingly, fewer than 30% of radiomic models undergo any form of external validation, and only a small minority validate across multiple institutions, countries, or scanner platforms [134]. When externally validated, model performance typically declines sharply, with AUC reductions often exceeding 0.15 compared with internal validation results [135, 136]. This significant performance decay arises primarily from discrepancies in patient demographics, disease spectrum, scanner manufacturers, imaging parameters, and reconstruction protocols. In the pan‐cancer setting, these differences are further amplified by tissue‐specific characteristics, anatomical backgrounds, and tumor microenvironment diversity, making cross‐cancer generalization exceptionally challenging [137].

Compounding these issues is the widespread lack of standardized data acquisition, processing, and reporting [138, 139]. Many studies fail to document key scanning parameters, use inconsistent segmentation protocols, or lack transparent inclusion/exclusion criteria [140, 141, 142, 143]. Without rigorous and reproducible data pipelines, radiomic features cannot be reliably compared or integrated across studies, resulting in fragmented evidence that cannot support broad clinical implementation.

### 5.2 Domain Shift Across Cancers, Scanners, and Protocols

Domain shift—the discrepancy in data distribution between training and target populations—is particularly pronounced in pan‐cancer radiomics [144, 145]. Unlike single‐disease applications, pan‐cancer radiomics must contend with biological heterogeneity across tumor types, technical heterogeneity across imaging devices, and procedural heterogeneity across clinical protocols. These sources of shift collectively undermine model robustness and generalizability [146].

Biological domain shift arises from inherent differences between tumor types, including tissue density, vascularity, cellularity, stromal composition, and anatomical background [147]. For example, lung tumors are surrounded by air‐filled parenchyma, whereas liver tumors exist within a highly perfused and heterogeneous organ background, and brain tumors are influenced by cerebrospinal fluid and edema [148]. These tissue‐specific environments alter radiomic feature profiles and reduce cross‐cancer model applicability.

Technical domain shift stems from variations in CT, MRI, and PET systems from different manufacturers, along with differences in tube voltage, slice thickness, reconstruction kernel, magnetic field strength, and contrast injection protocols [149, 150]. Even minor variations can induce 15%–20% variability in radiomic feature values, severely compromising model stability.

Protocol domain shift reflects differences in clinical workflow, including timing of imaging relative to treatment, contrast timing, patient positioning, and radiologist‐dependent interpretation [151]. Together, these forms of domain shift create a formidable barrier to developing universal radiomic models that perform reliably across institutions, platforms, and cancer types.

### 5.3 Reproducibility Crisis and the Need for Prospective Pan‐Cancer Trials

Radiomics as a field faces a growing reproducibility crisis, driven by inadequate reporting standards, insufficient quality control, and a lack of large‐scale prospective investigations [152]. Many published studies use non‐standardized feature extraction software, fail to report segmentation variability, and omit key methodologic details, making independent replication nearly impossible.

Although reporting guidelines such as STARD‐RAD, CLAIM, and TRIPOD‐AI have been developed, their adoption remains low. Similarly, quality assessment tools including RQS, QUADAS‐2, and PROBAST are underutilized, leading to the publication of methodologically weak studies with limited clinical value. This high volume of low‐reproducibility research dilutes the overall evidence base and erodes clinician confidence.

A critical deficiency in the field is the near absence of prospective, multi‐center, pan‐cancer clinical trials designed specifically to validate radiomic models [153]. Nearly all available evidence is derived from retrospective analyses, which are prone to bias, unmeasured confounding, and overfitting [154]. Without prospective data demonstrating clinical utility, improved patient outcomes, and cost‐effectiveness, radiomics cannot be recommended in clinical guidelines or be widely adopted.

The lack of coordinated prospective efforts also delays the establishment of standardized radiomic panels, consensus thresholds, and decision‐making pathways [155]. For radiomics to advance beyond a research tool, large coordinated consortia are needed to conduct prospective validation across multiple cancer types, imaging platforms, and healthcare systems.

### 5.4 Regulatory Pathways (FDA, NMPA, CE) for Cancer Radiomics Devices

Regulatory uncertainty represents a major bottleneck for the clinical translation of radiomics tools [156]. Regulatory frameworks for artificial intelligence and radiomics products are still evolving globally, with distinct requirements across the United States (FDA), China (NMPA), and Europe (CE). In all three regions, radiomic AI tools lack clear, disease‐specific classification standards, leading to ambiguity in risk stratification, validation endpoints, and clinical utility evidence requirements.

FDA has implemented software‐as‐a‐medical‐device (SaMD) pathways, with a focus on premarket notification (510(k)) and De Novo classification for low‐to‐moderate risk AI tools [157, 158]. NMPA has established AI medical device approval channels but requires stringent multi‐center prospective validation and real‐world data. CE marking follows a self‐certification model but demands compliance with essential health and safety requirements. Despite these pathways, none provide fully tailored guidelines for pan‐cancer radiomic biomarkers that integrate imaging quantification and machine learning.

**FIGURE 5:**
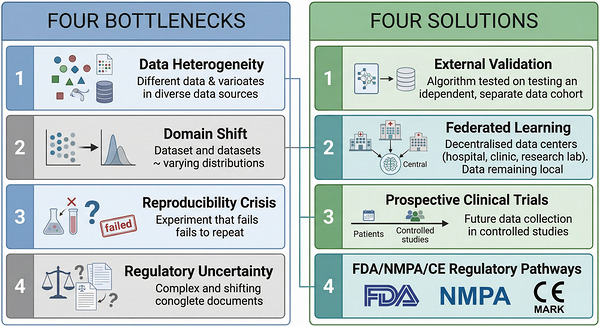
Major bottlenecks and corresponding solutions for clinical translation of pan‐cancer radiomics. Key obstacles include data heterogeneity, domain shift across cancers and scanners, reproducibility crisis, and unclear regulatory pathways. Practical solutions involve large‐scale external validation, federated learning for privacy‐preserving collaboration, prospective multi‐center trials, and compliance with FDA, NMPA, and CE regulatory frameworks.

Regulatory approval universally requires robust evidence of safety, effectiveness, reproducibility, and clinical value [159]. However, very few radiomics models have completed the large‐scale, prospective, multi‐center testing necessary for regulatory submission [160]. Post‐market surveillance, continuous learning protocols, and long‐term safety monitoring also remain poorly defined, discouraging industrial investment and clinical deployment. Consequently, only a small number of radiomics‐related products have obtained marketing authorization for oncology indications, leaving most academic advances trapped in the translational gap between research and clinical practice (Figure 5).

## 6 Future Directions: Toward Generalizable and Causal Radiomics

The future of radiomics lies in the development of generalizable, causal, interpretable, and clinically integrated models that can be reliably deployed across institutions, platforms, and cancer types [161]. Driven by advances in artificial intelligence, multi‐center collaboration, and multi‐omics integration, radiomics is poised to evolve from a retrospective research technique into a real‐time clinical decision‐support tool.

### 6.1 Foundation Models for Multi‐Cancer Radiomics

Foundation models—large‐scale neural networks pre‐trained on massive, diverse datasets—represent a transformative advance for pan‐cancer radiomics [162]. Unlike traditional models trained on single tasks or single cancer types, foundation models learn generalizable visual and semantic representations from millions of images across multiple diseases, modalities, and anatomical sites.

Once pre‐trained, these models can be efficiently adapted to new tasks and new cancer types using only small datasets, enabling rapid development and deployment [163]. For radiomics, foundation models can unify feature learning across CT, MRI, and PET, reduce domain shift, and improve performance in rare tumors or limited‐data settings. By learning universal imaging patterns across cancer types, foundation models inherently support cross‐cancer generalization and reduce the impact of technical and biological heterogeneity.

Complementary to foundation models are vision‐language models, which jointly learn imaging and text data [164]. By integrating radiological images with clinical reports, pathology descriptions, and molecular results, vision‐language models enhance interpretability, enable natural‐language queries, and support more intuitive clinical integration. Together, foundation models and vision‐language models will enable truly universal radiomics systems that generalize across cancer types [165].

### 6.2 Causal Inference for Treatment‐Effect Heterogeneity Across Cancers

A key limitation of conventional radiomics is its reliance on correlational rather than causal relationships [166]. Most models identify statistical associations between features and outcomes but cannot determine whether a feature directly influences treatment response or is merely a bystander [167, 168, 169]. Causal inference methods—including Mendelian randomization, causal forests, and inverse probability weighting—address this gap by estimating genuine causal effects.

In pan‐cancer radiomics, causal inference can be used to identify radiomic features that directly predict treatment benefit, rather than simply prognosticating outcome [170, 171]. This allows clinicians to distinguish between patients who benefit from a specific treatment and those who do not, enabling truly personalized oncology.

Causal methods also reduce confounding and improve model transportability across populations and cancer types [172]. By focusing on causal mechanisms rather than spurious correlations, causal radiomic models are more robust to domain shift, more interpretable, and more suitable for clinical trust and adoption [173].

### 6.3 Federated Learning Enabling Multi‐Center, Multi‐Cancer Collaborations

Federated learning has emerged as a pivotal technology to overcome data scarcity, privacy restrictions, and regulatory barriers in pan‐cancer radiomics [174]. In conventional centralized studies, data must be transferred to a central server, raising privacy risks and regulatory burdens. Federated learning instead allows multiple institutions to collaboratively train a shared model without transmitting raw patient data, following the principle of “model moves, data stays.”

This approach enables the construction of extremely large, diverse, pan‐cancer datasets while complying with privacy regulations such as GDPR and HIPAA [175]. Federated learning improves model generalization, reduces domain shift, and accelerates external validation. It also facilitates international collaboration across healthcare systems, which is essential for developing universally applicable radiomic tools.

In the future, federated learning will be combined with blockchain, differential privacy, and homomorphic encryption to further enhance security and accountability [176, 177, 178]. This ecosystem will enable the development of next‐generation pan‐cancer radiomics models trained on tens of thousands of patients across dozens of institutions.

### 6.4 Integration Into Clinical Workflows and RCTs

For radiomics to reach routine clinical use, it must be seamlessly integrated into existing clinical infrastructure, supported by high‐quality evidence from randomized controlled trials (RCTs), and embedded within clinical guidelines [179].

First, radiomics analysis must be implemented as lightweight, real‐time modules within picture archiving and communication systems (PACS), providing automated segmentation, feature extraction, and model inference with analysis times under 5 min [180, 181]. User‐friendly interfaces with clear visualizations and interpretability tools will increase adoption by radiologists and oncologists.

Second, clinical translation requires prospective RCTs demonstrating that radiomics‐guided strategies improve survival, quality of life, or cost‐effectiveness compared with standard care [182, 183]. Such trials are currently rare but essential for guideline inclusion and regulatory approval.

Third, radiomics should be integrated into intraoperative and adaptive radiotherapy workflows, enabling real‐time surgical guidance, margin assessment, and dose adaptation [184]. Real‐time radiomics can improve resection precision, reduce recurrence, and minimize toxicity.

**FIGURE 6:**
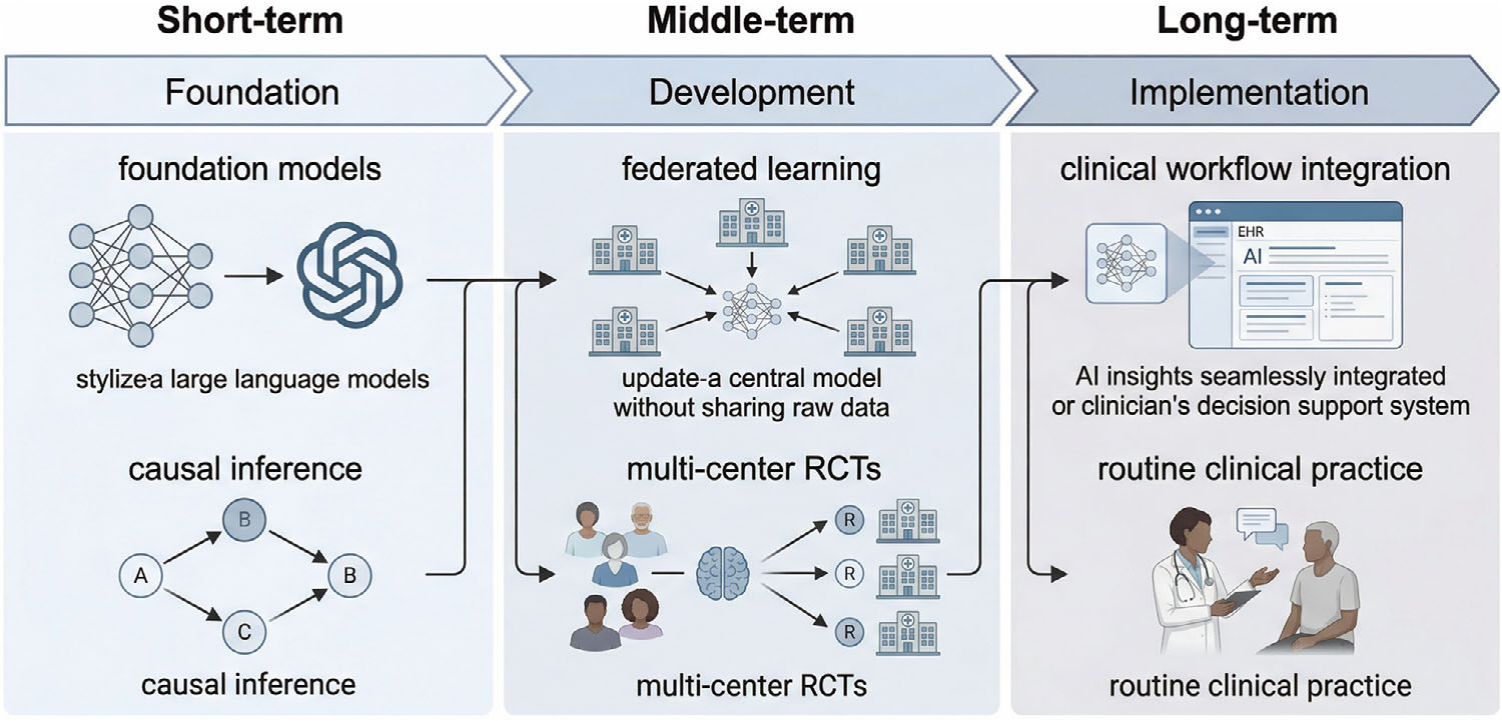
Future roadmap toward generalizable and clinically actionable pan‐cancer radiomics. Short‐term development focuses on foundation models and causal inference. Middle‐term advances include federated learning and multi‐center randomized controlled trials. Long‐term goals involve seamless integration into routine clinical workflows, regulatory approval, and widespread clinical implementation.

Fourth, sustainable clinical implementation requires clear educational programs, inclusion into clinical practice guidelines, and established reimbursement pathways [185]. As radiomics accumulates high‐quality evidence from RCTs and real‐world studies, it will gradually become a standard component of precision oncology practice (Figure 6).

## 7 Conclusion and Outlook

Radiomics has evolved from a conceptual technical framework to a promising and versatile pan‐cancer precision tool that covers the full continuum of oncology care from early detection to prognostic stratification [186]. Technically, a unified and standardized pipeline has been established with rational cancer‐specific adaptations [187]. Advanced branches including delta radiomics and habitat radiomics, together with multi‐omics integration, have greatly enhanced biological interpretability and clinical relevance. Clinically, radiomics demonstrates consistent and valuable performance across solid tumors in early diagnosis, molecular prediction, treatment monitoring, and prognosis stratification, with lung cancer representing the most mature and robust paradigm [188].

However, pan‐cancer radiomics still faces three core bottlenecks that limit broad clinical implementation [189, 190]: insufficient technical standardization leading to multi‐source heterogeneity, inadequate clinical validation dominated by retrospective single‐center studies, and limited model interpretability and reproducibility. These challenges restrict cross‐cancer generalization and real‐world utility.

Future efforts must focus on promoting universal IBSI‐aligned standardization protocols, launching large‐scale prospective pan‐cancer trials, developing explainable and causal artificial intelligence models, deploying privacy‐preserving federated learning for multi‐center collaboration, and driving systematic integration into clinical workflows and regulatory pathways. Distinguishing between generalizable principles and cancer‐specific characteristics will be key to maximizing translational value.

With the integration of foundation models, causal inference, multi‐omics, and real‐world evidence, radiomics will transform from a research tool into a clinically actionable precision oncology platform [191]. Ultimately, pan‐cancer radiomics will enable noninvasive, dynamic, and truly individualized clinical decision‐making, significantly improving survival and quality of life for patients across multiple solid tumor types.

## Author Contributions

Jiangbo Shao wrote the manuscript, designed the figures, collected references, and edited the manuscript. Meng Wei and Ke Li conceived and revised the manuscript. Guangchao Lv, Kai Liu, and Ye Guo revised the manuscript and provided general guidance. All authors approved the final version.

## Ethics Statement

The authors have nothing to report

## Conflicts of Interest

The authors declare no conflicts of interest.

## Data Availability

Data supporting the findings are available from the corresponding authors upon reasonable request.
